# Ectopic expression of *TaBG1* increases seed size and alters nutritional characteristics of the grain in wheat but does not lead to increased yields

**DOI:** 10.1186/s12870-021-03294-x

**Published:** 2021-11-10

**Authors:** Matthew J. Milner, Sarah Bowden, Melanie Craze, Emma J. Wallington

**Affiliations:** NIAB, 93 Lawrence Weaver Road, Cambridge, CB3 0LE UK

**Keywords:** Seed size, Big grain 1, Nitrogen, Zinc, Iron, Seed number, Auxin

## Abstract

**Background:**

Grain size is thought to be a major component of yield in many plant species. Here we set out to understand if knowledge from other cereals such as rice could translate to increased yield gains in wheat and lead to increased nitrogen use efficiency. Previous findings that the overexpression of *OsBG1* in rice increased yields while increasing seed size suggest translating gains from rice to other cereals may help to increase yields.

**Results:**

The orthologous genes of *OsBG1* were identified in wheat. One homoeologous wheat gene was cloned and overexpressed in wheat to understand its role in controlling seed size. Potential alteration in the nutritional profile of the grains were also analyzed in wheat overexpressing TaBG1. It was found that increased TaBG1-A expression could indeed lead to larger seed size but was linked to a reduction in seed number per plant leading to no significant overall increase in yield. Other important components of yield such as biomass or tillering did not change significantly with increased *TaBG1-A* expression. The nutritional profile of the grain was altered, with a significant decrease in the Zn levels in the grain associated with increased seed size, but Fe and Mn concentrations were unchanged. Protein content of the wheat grain also fell under moderate N fertilization levels but not under deficient or adequate levels of N.

**Conclusions:**

TaBG1 does control seed size in wheat but increasing the seed size *per se* does not increase yield and may come at the cost of lower concentrations of essential elements as well as potentially lower protein content. Nevertheless, *TaBG1* could be a useful target for further breeding efforts in combination with other genes for increased biomass.

**Supplementary Information:**

The online version contains supplementary material available at 10.1186/s12870-021-03294-x.

## Background

Improving crop yield while maintaining levels of inputs such as nitrogen (N) fertilizer, is necessary to ensure food security for the projected increase in population in years to come. Wheat (*Triticum aestivum* L.) is one of three main staple crops worldwide grown on every continent, except Antarctica, and is planted on over 200 million hectares annually [1]. Unfortunately wheat yields in many parts of the world have plateaued since the mid-1990 s [2]. This yield plateau can be attributed partly to inadequate agronomic practices, however with the release of an increasingly better reference wheat genome, gains from reverse genetics shown in other model species or crops can now be translated to wheat [3–6]. More recent progress in yield enhancement in wheat has been attributed to the increase in spikelet fertility, or the number of kernels per spikelet. This led to the number of seeds per spike and spikelet fertility also being correlated with increased yield [7]. Unfortunately, in wheat both seed number and spikelet fertility showed a negative correlation with thousand-grain weight [7]. Combining both spikelet fertility and seed size may help to drive further yield enhancements. In wheat, since single grain weight varies depending on growth conditions, yield is affected by both the grain number and size [8, 9]. Thus, the targets for a high yield may be more complicated in wheat than in rice where grain number is the major limitation to yield [10, 11]. Thus a greater understanding of components of grain size can lead to improvement in the genetic stock of the cultivars grown, leading to further yield improvements for farmers.

Grain size and weight, which is determined by its three-dimensional structure (length, width, and thickness) is a crucial component that affect yield, quality and nutrition [12]. In the context of crop domestication and artificial breeding, grain size and weight have always been important agronomic traits for selection. One of the major components of increasing grain yield in all crop species is increased seed size. In several plant species an increase in seed size led to increased yields either per plant or in the field. Advancements in our understanding of the genes and or regions of the genome involved in the control of grain size continues to increase. There are several genes which have been identified as playing a role in the modification of seed size which first were identified in rice and later translated to wheat as genetic resources became available. This includes modification of pathways involved in proteasomal degradation (GW2 and GW5/qSW5), phytohormone signaling (OsGS6, TGW6, OsBG1, and OsCKX2), and G protein-mediated signal transduction (GS3 and RGB1); which are thought to work by regulating cell division and/or cell expansion in specific grain tissues [13].

Several wheat genes have also now been isolated and suggested to be associated with thousand grain weight (TGW). These include TaCwi [14, 15], TaTGW6 [16, 17], TaGW2-A1 [18–20], TaGS1a [21], TaGS5-3 A [22], TaGASR7-A1 [23], TaPSTOL [24], TaFlo2 [25], TaCYP78A3 [26], and TaCYP78A5 [27], da1 [28]. Many of these genes have trade-offs with other yield components which ultimately limit their ability to increase yields alone. Increasing our knowledge of genes and pathways involved in seed size and shape are important to understand their role in yield prior to incorporation into breeding programs and the development of new higher yielding varieties.

Other considerations for the growth of a sustainable crop is the amount of inputs required and their efficient utilization by the crop. Nitrogen is one of the most yield-limiting nutrients in crop production, and a proper understanding of its role in yield components is essential for improving grain yield. In both rice and wheat, grain number is linearly correlated with total plant N content. It has been suggested that most of the variation in both grain number and yield is caused by differences in resource accumulation, particularly nitrogen, by the crop [29]. Application of nitrogen fertilizers prior to the panicle initiation stage in rice is known to affect inflorescence development and increase flower numbers per panicle which is a major driver of increasing yield [10]. There is evidence that rice and wheat may behave differently in their response to N fertilizer, including how they partition N in leaves of the canopy and its effect on yield [30]. This understanding has led to studies where modification of genes involved in increasing seed size does not always lead to an increase in yield in wheat [18, 19]. Other limitations in the translation of genes from rice to wheat is that the architecture of the rice inflorescence and wheat ear are quite different. Some of the genes which have been shown to alter grain size and shape, such as GS3, which has a multi-domain structure, can change the effect it has on seed size and plant height, depending on where the mutations are located [31–33]. Other genes may also alter many potential yield components in addition to seed size, or be specific to the way the crop matures and if the crop has an indeterminate growth habit [33, 34]. Few characterized genes which increase seed size, positively impact on overall yield without pleiotropic effects.

Of major concern is the potential for a reduction in nutritional quality of the grain with increased grain size. As most of the micronutrients are located in the aleurone layer, a change in the endosperm to aleurone layer ratio can reduce the nutritional quality of the consumed food product. This is of serious concern in many countries including the UK, where over half of girls aged 11 to 18 are considered anemic [35]. Thus a reduction in the amount of Fe in the grain could reduce intakes further in an already Fe deficient population. Indeed over the past sixty years the levels of Fe, Zn, Cu and Mg in the grain have decreased significantly as yield has doubled, coinciding with the introduction of semi-dwarf, high-yielding varieties of wheat [36].

Wheat is also a large component of protein in the human diet, with cereals and cereal products providing more than 20 % of the protein in the diet in the UK for children over 1.5 years and adults [37]. It is also known that N fertilization levels can influence protein content with typical ranges of protein content ranging from 10 to 15 % but can be as high as 22 % in some wheat collections [38, 39]. If less than optimum N application is applied to a crop, a lower protein content or reduced yields would likely occur. This reduction in protein or yield can have both a nutritional and economic costs affecting overall human health.

To understand the role increased seed size could have on yield and nutritional traits we studied the effects of increased *TaBG1* expression in wheat and its role to shape seed size, yield components, and nutritional qualities.

## Methods

### Gene identification

The *OsBG1* coding sequence from NCBI (Q10R09.1) also known as Os03g0175800 was used as a query for Big Grain genes in Ensemble plants. (http://plants.ensembl.org/index.html). Protein domain identification was found using the biomart section of Ensembl plants v42 to search both the rice and wheat genomes for genes using the Interpro ID IPR039621. All DNA and Proteins sequences are available at Ensembl plants. Expression profiles of the Big Grain like genes was downloaded from the Wheat Expression Browser, (http://www.wheat-expression.com) [40]. Phylogenetic tree was constructed using the amino acid sequences of the Big Grain genes in MEGA X [41]. Sequences were aligned using the MUSCLE algorithm and the tree was constructed using 500 iterations using the Maximum Likelihood Tree option. Heat map of expression values was constructed using heatmapper (http://www.heatmapper.ca/) [42].

### Wheat transformation

 Wheat variety Fielder (USDA, available from CIMMYT, under the name BW 35215) plants were grown in controlled environment chambers (Conviron) at 20^°^C day/15^°^C night with a 16 h day photoperiod (approximately 400 µE m^− 2^ s^− 1^). Immature seeds were harvested for transformation experiments at 14–20 days post-anthesis (dpa). Isolated immature wheat embryos were co-cultivated with *Agrobacterium tumefaciens* for 2 days in the dark [43]. Subsequent removal of the embryonic axis and tissue culture was performed as described by [44]. Individual plantlets were hardened off following transfer to Jiffy-7 pellets (Jiffy), potted up into 9 cm plant pots containing M2 and grown on to maturity and seed harvest in controlled environment chambers, as above.

### Plasmid construction for genetic modification

*TaBG1-A* was synthesized from the public sequence available at the time for the wheat cultivar Chinese Spring with attL1 and attL2 sites for direct recombination into binary gateway vectors. *TaBG1-A* was then recombined into the binary vector pSc4ActR1R2 using a Gateway LR Clonase II Kit (Thermofisher) to create pMM14. *TaBG1-A* was expressed in wheat cv Fielder from the rice *Actin* promoter and transcripts terminated by the *Agrobacterium tumefaciens* nopaline synthase terminator (tNOS).

pMM14 was verified by restriction digest and sequencing before being electro-transformed into *A. tumefaciens* strain EHA105. Plasmids were re-isolated from *Agrobacterium* cultures and verified by restriction digest prior to use in wheat experiments [45].

### DNA analysis of transformed wheat plants

 Plantlets which regenerated under G418 selection in tissue culture were transferred to Jiffy-7 pellets and validated using an *nptII* copy number assay relative to a single copy wheat gene amplicon, GaMyb, normalized to a known single copy wheat line. Primers and Taqman probes were used at a concentration of 10µM in a 10 µl multiplex reaction using ABsolute Blue qPCR ROX mix (Thermofisher) with the standard run conditions for the ABI 7900 HT. The relative quantification, ^ΔΔ^CT, values were calculated to determine *nptII* copy number in the T_0_ and subsequent generations [46]. Homozygous transgenic lines were identified on the basis of *nptII* copy number and segregation analysis. WT Fielder plants were null segregants.

### Expression of TaBG1 in wheat

Total RNA was isolated from wheat shoots using a RNeasy Kit (Qiagen). Following treatment with DNaseI (Thermofisher), cDNA synthesis was conducted on 500 ng of total RNA using Omniscript RT Kit (Qiagen). The cDNA was diluted 1:2 with water and 0.5 µL was used as template in each RT-PCR reaction. Transcript levels were quantified using SYBR Green JumpStartTaq ReadyMix (SIGMA) with the standard run conditions for the ABI 7900 HT. Three technical replicates were performed on each of the three biological replicates. Two reference genes *TaUbiquitin* and TaEF1α were used for the normalization using the ^ΔΔ^CT. The sequence of primers used in Q-PCR assays are *TaUbiquitin* F (5’-CCTTCACTTGGTTCTCCGTCT-3’), *TaUbiquitin* R (5’-AACGACCAGGACGACAGACACA-3’), TaEF1α F (5’-TGGTGTCATCAAGCCTGGTATGGT-3’), TaEF1α R (5’-ACTCATGGTGCATCTCAACGGACT-3’), TaBG1 F (5’-GCTGCTGGACGCGATATAC-3’) and TaBG1 R (5’-CTGCTTCTTGGCCTTCTTCT-3’).

### Growth conditions

 Transgenic lines and corresponding null segregants were grown on TS5 low fertility soil to control total nitrogen with a starting nitrogen level of 0.1 mg/l (Bourne Amenity, Kent, UK). Ammonium nitrate was then added to each pot to reach a final concentration in the pots equivalent to field fertilizer application of 70, 140 or 210 kg N /ha. Plants were grown in a climate controlled glasshouse with 10,000 lx sodium supplemental light for a 16 h day and 20^°^C/15^°^C day night temperatures. At least 21 plants per line were grown per treatment in a randomized design.

### Grain cross sections

 The wheat grains were soaked in distilled water and subjected to a vacuum system for 10 min. The seeds were then fixed overnight with 3 % EM grade glutaraldehyde in 0.1 M Cacodylate buffer and oscillated overnight at room temperature. Seeds were washed twice in 0.1 M cacodylate buffer for 10 min for each wash and then stored in 0.1 M Cacodylate buffer prior to post tissue-fixation with 1 % Osmium Tetroxide in 0.1 M Cacodylate Buffer for two days at 4 °C.

Seeds were then washed twice in distilled water for 10 min each time and dehydrated with 50 %, 70 %, 90 % (2 × 15 min for each) and 100 % ethanol (3 × 30 min) respectively. To further remove water from the samples, propylene oxide (100 %) was added (2 changes for 30 min at room temperature) followed by the addition of propylene oxide: resin (48 g TLV Resin, 16 g VH1 Hardener, 36 g TLV Hardener VH2) solution at 3:1 ratio (1 h, room temperature) and 1:1 ratio (overnight with lids off, room temperature) respectively. A pure resin preparation (TAAB Low Viscosity Resin – medium recipe) was then added (2 changes, 2 h per change) prior to embedding in the oven at 60 °C for 48 h. Samples were sectioned with a Histo diamond knife at 1000nm (1micron) thick using Leica EM UC6 (Leica Biosystems, Wetzlar, Germany), placed on APES coated slide and stained using 1 % Toluidine Blue in 1 % Sodium Borate. Sections were imaged using ZEISS Axiophot microscope and captured using Micromanager Software. Cell area was measured by tracing individual cells taken from images taken at 200X magnification using ImageJ [47].

### Elemental analysis

Sample digestates were diluted 1-in-10 using Milli-Q water prior to elemental analysis. The concentrations of 28 elements were obtained using inductively coupled plasma-mass spectrometry (ICP-MS; Thermo Fisher Scientific iCAPQ, Thermo Fisher Scientific, Bremen, Germany); Ag, Al, As, B, Ba, Ca, Cd, Cr, Co, Cs, Cu, Fe, K, Mg, Mn, Mo, Na, Ni, P, Pb, Rb, S, Se, Sr, Ti, U, V, Zn. Operational modes included: (i) a helium collision-cell (He-cell) with kinetic energy discrimination to remove polyatomic interferences, (ii) standard mode (STD) in which the collision cell was evacuated, and (iii) a hydrogen collision-cell (H2-cell). Samples were introduced from an autosampler incorporating an ASXpress™ rapid uptake module (Cetac ASX-520, Teledyne Technologies Inc., Omaha, NE, USA) through a PEEK nebulizer (Burgener Mira Mist, Mississauga, Burgener Research Inc., Canada). Internal standards were introduced to the sample stream on a separate line via the ASXpress unit and included Sc (20 µg L-1), Rh (10 µg L-1), Ge (10 µg L-1) and Ir (5 µg L-1) in 2 % trace analysis grade HNO_3_ (Fisher Scientific UK Ltd). External multi-element calibration standards (Claritas-PPT grade CLMS-2; SPEX Certiprep Inc., Metuchen, NJ, USA) included Ag, Al, As, B, Ba, Cd, Ca, Co, Cr, Cs, Cu, Fe, K, Mg, Mn, Mo, Na, Ni, P, Pb, Rb, S, Se, Sr, Ti (semi-quant), U, V and Zn, in the range 0–100 µg L-1 (0, 20, 40, 100 µg L-1). A bespoke external multi-element calibration solution (PlasmaCAL, SCP Science, Courtaboeuf, France) was used to create Ca, K, Mg and Na standards in the range 0–30 mg L-1. Boron, P and S calibration utilized in-house standard solutions (KH_2_PO_4_, K_2_SO_4_ and H_3_BO_3_). In-sample switching was used to measure B and P in STD mode, Se in H2-cell mode and all other elements in He-cell mode. Sample processing was undertaken using Qtegra™ software (Thermo Fisher Scientific) with external cross-calibration between pulse-counting and analogue detector modes when required.

### N measurements

 Samples were measured using the Dumas method for N determination. The samples were dried for 17 h at 100 °C and then milled on a 1mm hammer mill. Prior to testing the sample were dried at 104 °C for 3 h and 1 g of sample was loaded on the instrument (Leco TruMacN Dumas gas analyser), following the manufacturer’s instructions. Samples were converted to gases by heating in a combustion tube at 1150 °C. Interfering components are removed from the resulting gas mixture. The nitrogen compounds in the gas mixture or a representative part of the mixture, are converted to molecular nitrogen which is quantitatively determined by a thermal conductivity detector. The nitrogen content is then calculated by a microprocessor. To calculate the percent protein the standard ISO conversion factor of 5.7 was applied [48].

### Statistical analyses

Analysis of variance (ANOVAs) or Wilcox Tests were run using the aov and TukeyHSD functions in the R environment with the null hypothesis of no difference between lines [49]. Tukey’s post hoc test was added to identify each significant interaction between the lines tested. Data was plotted using R ggplot2 [50].

## Results

### Identification of BG1 orthologues in wheat

**Fig. 1:**
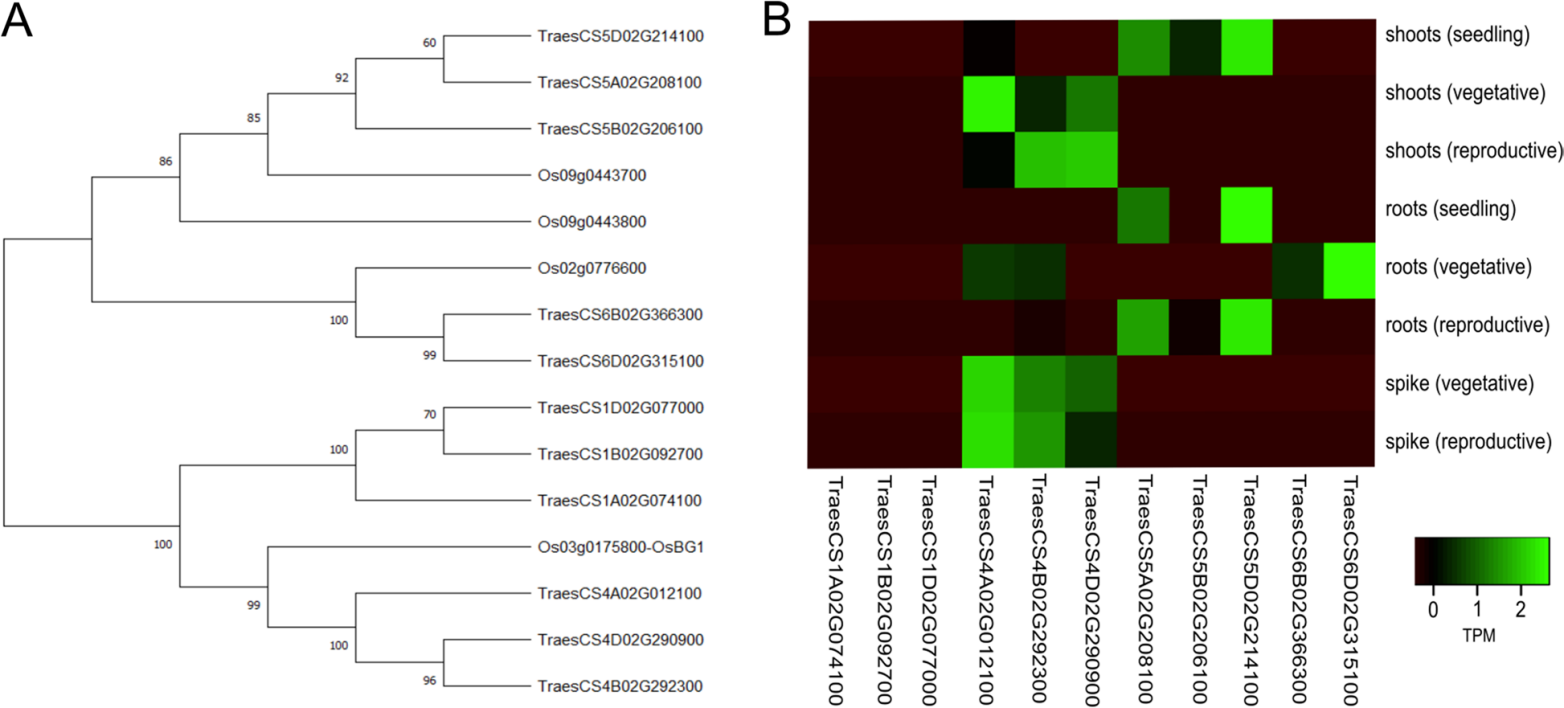
Comparison of rice and wheat BG1 genes. **A** Phylogenic tree of BG1-like genes in rice and wheat. The sequence identifiers correspond to corresponding gene models based on the RefSeq v1 wheat genome. **B** Expression of BG1-like genes in wheat. Expression values are shown in the heatmap as transcripts per million (TPM). Data shown is taken from wheat-expression.com for Chinese Spring without additional stress at the major tissue level

 To identify *OsBG1* orthologues in wheat the amino acid sequence for Interpro ID IPR039621 was used in a BlastP search against the Ensembl v 42 database. In rice there are four *BG1* like genes but in wheat eleven genes containing the big grain domain were identified (Fig. 1 A). Alignment of the rice and wheat BG1 like proteins reveals two rice genes Os09g0443700 and Os090443800 cluster with a set of genes located on chromosome 5, a third rice gene (Os02g077660) clusters with a pair of genes on chromosome 6 and the final rice gene OsBG1 (Os03g0175800) clusters with a set of gene on chromosome 1. The homoeologous gene triads which are closely related to OsBG1 located on wheat chromosomes 1 and 4. However alignments of the predicted amino acid sequences suggest that the homoeologous genes on chromosome 4 share 73 to 76 % identity whereas the chromosome 1 homoeologues shared 51–52 % identity with OsBG1 at the amino acid level (Suppl. Table 1). To further narrow down an ortholog of *OsBG1*, the expression profiles of the eleven *BG1-like* genes from wheat in public expression databases were examined. This indicated that the genes located on chromosome 4 are the most highly expressed of the family members. The homoeologous genes on chromosome 4 were expressed later in development and not during the seedling stage of growth and were the only genes to show expression in the spike tissues. This contrasts with the genes showing weaker expression located on chromosome group 5 mainly expressed during the seedling stage in both the roots and shoots but also in the root during reproduction/flowering. The 5B homoeologue does not appear to be expressed in any of the main tissues. The homoeologues on chromosome group 6 were only expressed in the roots during the vegetative growth portion of the life cycle. While current gene models do not show a direct homoeologue on chromosome 6A, the lack of a homoeologous gene on 6A might be an artifact of the current gene model prediction as a gene model appeared to exist in previous versions of the genome under the gene model TraesCS6A02G526800LC, but is now not found in version Ref Seq v1.1 of the wheat genome. No observable expression of the genes located on chromosome 1 in the tissues was identified (Fig. 1B). It should also be noted that of the three sets of genes on chromosomes 4, 5 and 6 the homoeologous set of genes are expressed at different levels and sometimes in different tissues suggesting some type of differential regulation of the BG family in wheat. The unique expression of the chromosome 4 homoeologues in the spike tissue led to the further characterization of the putative orthologs on chromosome 4 for further study. As the homeologue on 4A is expressed the highest, based on publicly available expression, the homeologue of 4A was specifically tested for overexpression studies.

### **Overexpression of*TaBG1-A*in wheat**

**Fig. 2:**
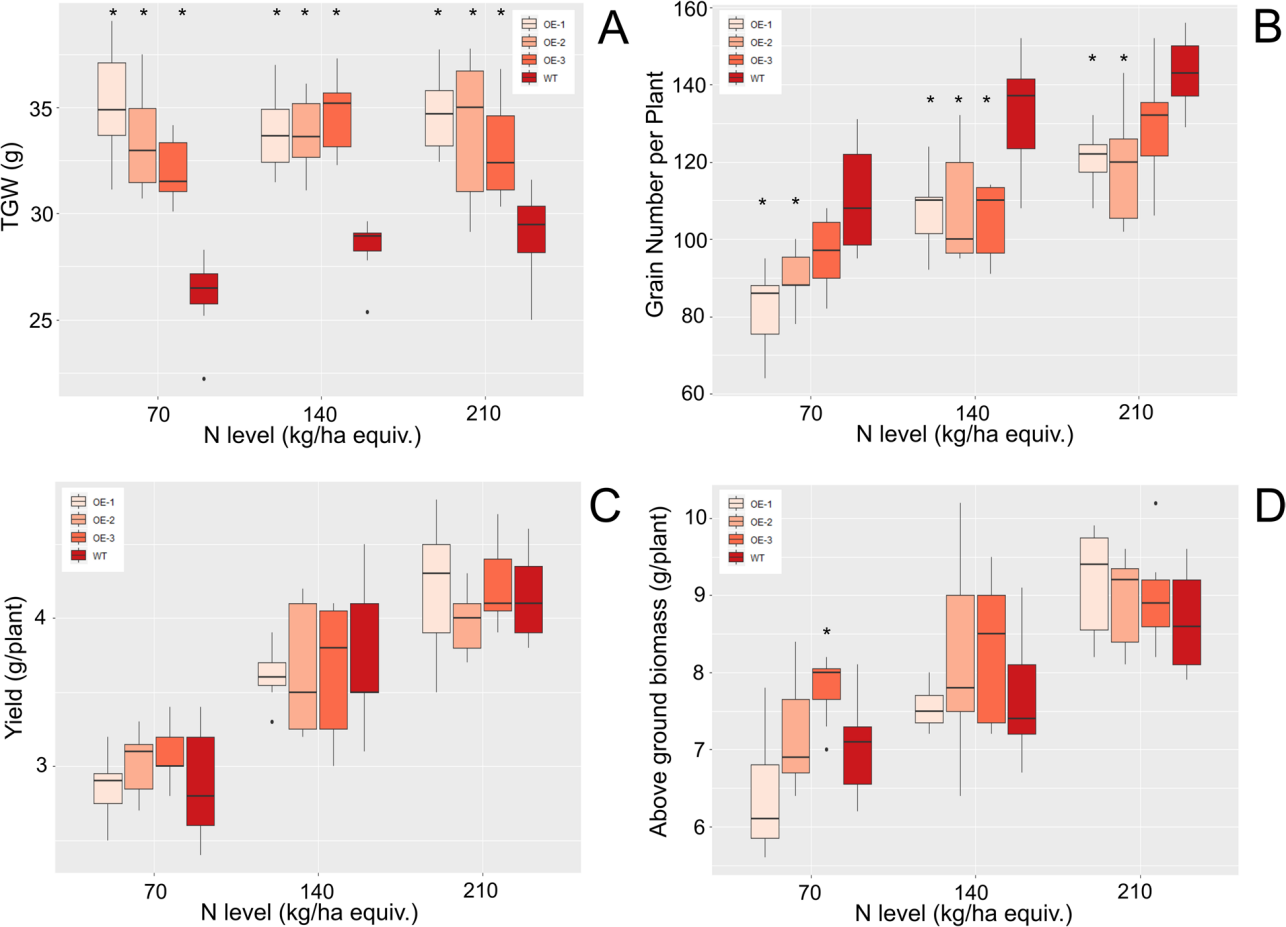
Agronomic measurements of the overexpression of *TaBG1-A.* Shown are the measurements from twenty-one plants per treatment under three levels of nitrogen, low (70), sufficient (140), and high (210) levels of N. **A** Seed weight (TGW) **B **Grain number per plant **C** Yield per plant **D** Above ground biomass per plant. Significant differences are shown by * which represent a p val. < 0.05 relative to WT Fielder

To understand if the genes on chromosome 4 could be the functional orthologues of OsBG1 *TaBG1-A* was overexpressed in wheat from the constitutive promoter OsActin. A total of thirty-seven independent transgenic lines were regenerated and the number of T-DNAs inserted determined in each T_0_ plant measured by *nptII* copy number analysis. To limit the possible adverse effects of multiple insertions, nine single- and five two-copy T-DNA insertion lines were prioritized for *TaBG1* expression determination. Three independent single insertion lines with increased expression in the leaves of the T_0_ plants from 4.9 to 6.3-fold over endogenous expression of *TaBG1* in the leaf were taken forward for further analysis of grain and agronomic phenotypes (Suppl. Figure 1). These T_0_ lines (OE-1, OE-2 and OE-3) were then selfed and the T_1_ plants assayed for T-DNA copy number to identify lines homozygous for the T-DNA insertion, which were then grown to T_2_ seed for further analysis. Several agronomic traits were measured for differences between the overexpression lines and a null segregant hereto referred as WT. The three overexpression lines were grown on a low fertility soil with three different levels of N including a deficient (70 kg/ha equiv.), a moderate (140 kg/ha equiv.) and an adequate level of N (210 kg/ha equiv.) added to understand the role N may play in seed size. Average seed weight did not vary with changes in N level in any of the four lines tested, however all three overexpression lines showed significantly increased TGW than a segregated null line used as a control (Fig. 2 A). Yield did significantly increase with increasing N supplied in a dose dependent manner (p val < 0.01) but comparisons of the three overexpression lines to WT at each N level showed no significant differences (Fig. 2 C). When the overexpression lines were compared to control lines the increased TGW did not increase overall yields. The lack of an increase in yield was due to the significant decrease in the number of seeds per plant in the overexpression lines, (p val < 0.05), suggesting that the plant simply traded off seed number to increase seed size (Fig. 2B). Other potential agronomic traits such as tiller number, which we defined as the tillers which produced an inflorescence, or biomass also did not differ significantly between the overexpression lines and WT plants (Fig. 2D and Suppl. Figure 2). The fact that there were no significant differences in biomass, apart from one line OE-3 grown on 70 kg/ha N, and no significant differences in yield, then led to no change to the harvest index for any treatment (p val OE-1 = 0.98, OE-2 = 0.24, OE-3 = 0.13) (Suppl. Figure 3).

### Components of increased grain size

**Fig. 3:**
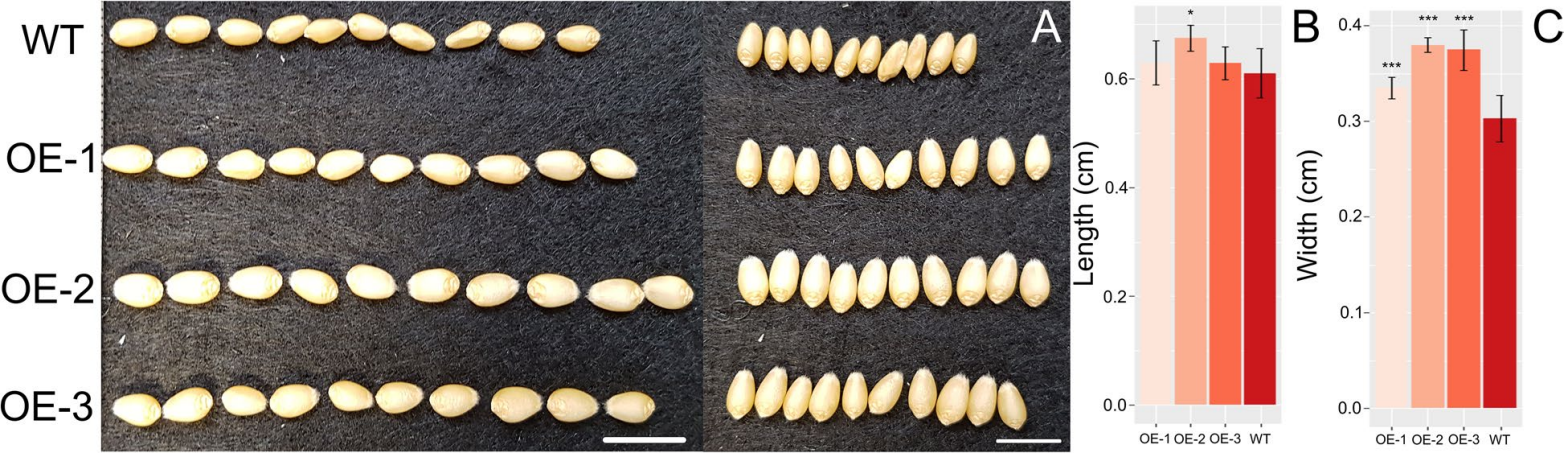
Comparison of seed length and width in *TaBG1-A* overexpression lines. **A** Ten seeds from the three independent *TaBG1-A* overexpression lines aligned to show differences in length and width relative to WT Fielder. Scale bar = 1 cm. **B** Average grain length in three independent *TaBG1-A* over expression lines relative to WT Fielder. **C** Average grain width in three independent *TaBG1-A* overexpression lines relative to WT Fielder. * = p val. < 0.05, *** = p val. <0.001 relative to WT Fielder

**Fig. 4:**
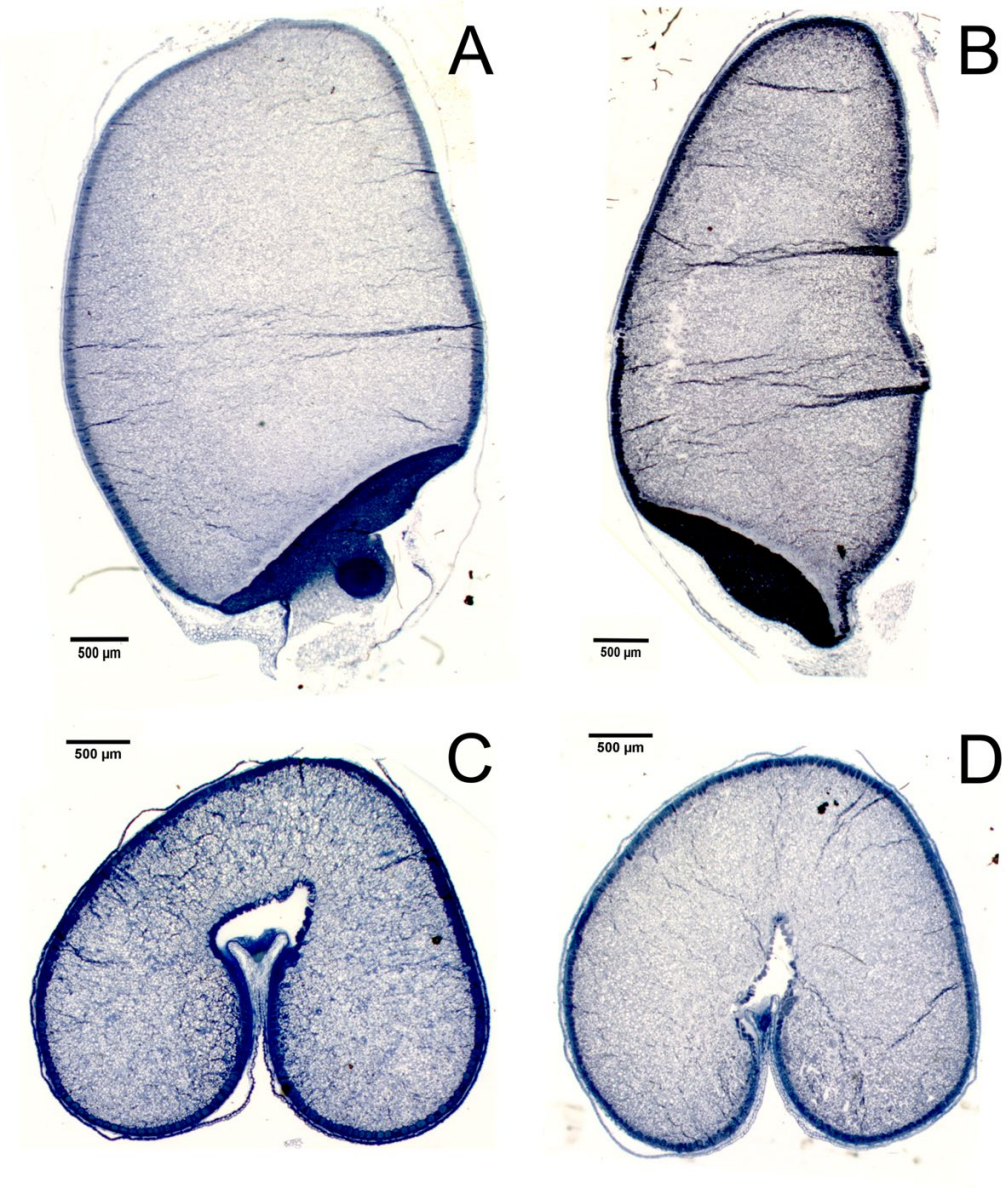
Longitudinal and transverse sections of *TaBG1-A* (OE-1) overexpression line and WT Fielder. **A** OE-1 longitudinal section **B** WT longitudinal section **C** OE-1 transverse section **D** WT transverse section. Scale bar = 500mm

**Fig. 5:**
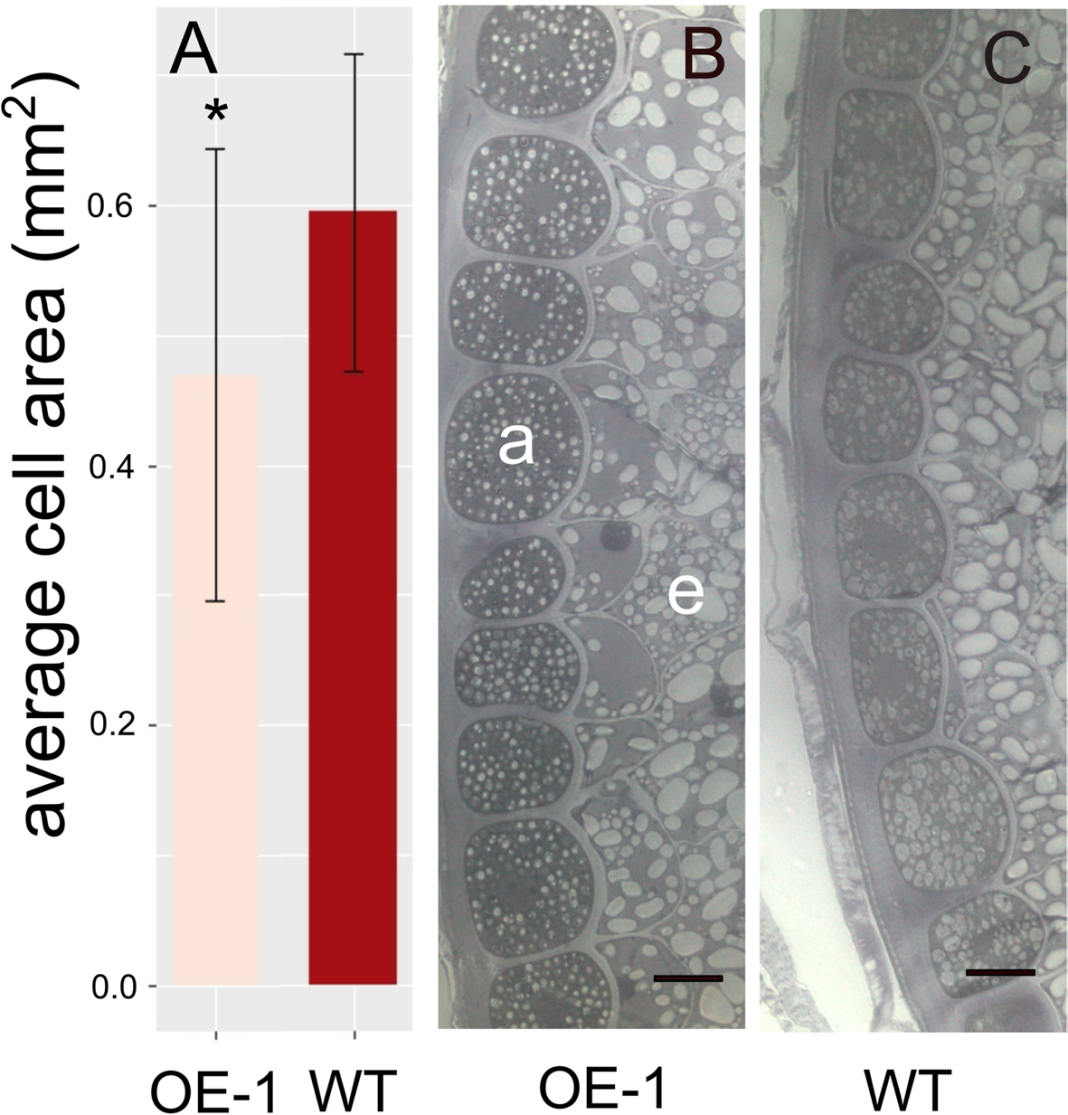
Comparison of cell size in the fully developed grain. **A** average aleurone cell size in OE-1 versus WT grains. Asterisk represents a significant difference (p val. 0.03). **B** and **C** representative images of OE-1 (**B**) and WT (**C**) aleurone and endosperm cells at 200X magnification. Scale bar = 0.1 mm, a signifies an aleurone cell and e signifies endosperm cells

 To understand which seed parameters were modified to increase the overall size of the wheat seeds the three OE lines were analyzed using MARVIN to measure the length and width components of grain size. From this it was found that on average the *TaBG1-A* overexpressing lines were marginally longer in length with only OE-2 showing significantly increased length (10 % greater than WT). While the other lines also appeared to show an increased average seed length, 3 % for both OE-1 and OE-3, the differences were not significant. Strikingly, seed width was significantly increased in the three OE lines with seeds of 10–26 % greater width than WT seeds, pval < 0.001 (Fig. 3). Cross sections of ten grains from line OE-1 and WT were also taken in two dimensions to understand the cause of these differences at a tissue level within the grain (Fig. 4). Differences were seen in the size of the aleurone layer cells with OE-1 having smaller and more numerous cells than WT grains (Fig. 5). OE-1 lines showed an average decrease of 21 % cell area relative to WT aleurone cells.

### Nutritional Content of TaBG1 overexpression lines

**Fig. 6:**
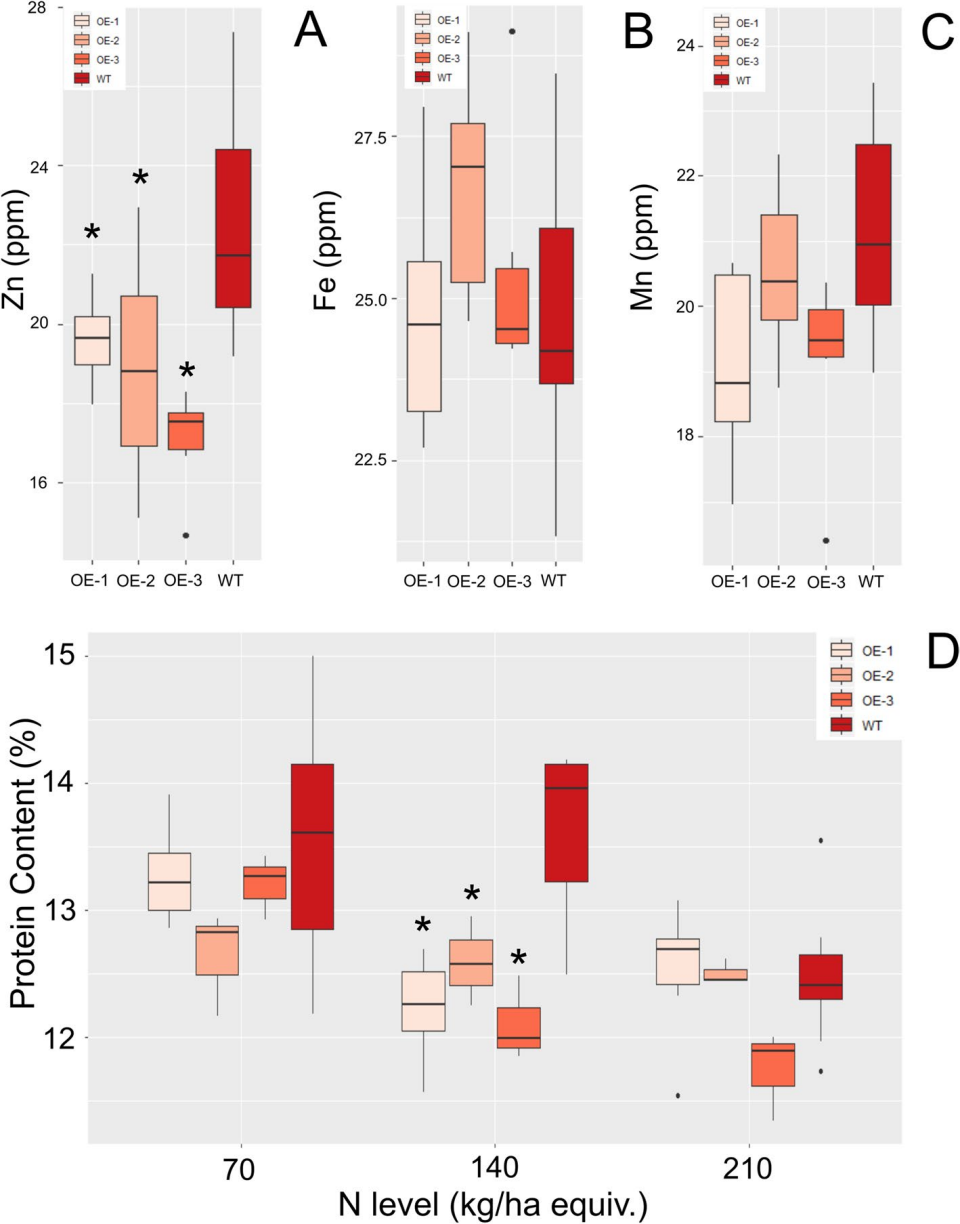
Micronutrient and protein content of *TaBG1-A *overexpression lines. **A** Zn, **B** Fe, **C** Mn, levels in grains of *TaBG1-A* overexpression lines relative to WT Fielder. **D** Protein content of *TaBG1-A* overexpression lines relative to WT Fielder grown on three different levels of N. * indicates a significant difference relative to WT p val. <0.05

 To understand the effect larger grain size could have on nutritional traits such as mineral content, grains were analyzed by ICP-MS to measure the mineral content (Fig. 6 A-C). All three of the lines tested showed significantly lower Zn levels in the grain relative to WT (p val, < 0.05, < 0.001, < 0.05). This contrasted with Fe and Mn levels as no significant differences were seen in these micronutrients. Other essential element concentrations were also altered in *TaBG1-A* overexpression lines, but only in two of the three lines tested; these include Ca, K and P (Suppl. Figure 4). One line OE-1 was also significantly lower in S (p val < 0.05). No differences were seen in any of the lines tested for Mg or B.

Nitrogen content of the grain was also determined from the plants grown under three different N fertilization levels using the Dumas method to measure the nitrogen content. As seen in Fig. 6D there were significantly lower levels of N in the grains of the OE lines versus WT when grown under a moderate level of N (140 kg/ha equiv.). There were no significant differences seen in the N content of the grain when grown on low or high levels of N. The lack of difference in yields also did not lead to any changes in nitrogen use efficiency (NUE) defined as yield divided by N concentration in the soil or, nitrogen efficiency ratio (NER) defined as yield divided by tissue N concentration at a given N concentration in the soil [51]. Using the nitrogen efficiency ratio there was a general trend towards a higher NER as lower N content in the grain but the p values ranged from 0.1 to 0.8 (Suppl. Figure 5).

## Discussion

A number of agronomic traits are believed to be involved increasing yield in plants. Grain size is one such trait and the target of numerous studies [14–27]. To understand the role in which increasing grain size can help to increase yields in wheat the orthologues of the rice Big Grain 1 (OsBG1) gene was identified in wheat and chosen for further characterization. BG1 was chosen as previous reports had shown that *OsBG1* could improve yields 15–20 % under field conditions with increased expression in rice [52]. The putative orthologues of OsBG1 in wheat identified on chromosome 4 were the only members of the 11 Big Grain like genes found in wheat to be expressed in the spike (Fig. 1). Increasing expression of the A homoeologue of *TaBG1* on chromosome 4 did in fact increase seed size with seeds on average 25 % larger by weight than a null segregant WT control (Fig. 2). This increase in grain size was mostly attributed to an increase in width and not to seed length, as seed length was only marginally increased (Figs. 3 and 4). At a seed tissue level cells in the mature grain of OE-1 showed a smaller average size than that of WT cells (Fig. 5). This average aleurone cell size difference was 21 % smaller in OE-1 grains relative to WT suggesting that the increase in size of the grain is due to increased endosperm cell number and or cell size. However, the increase in seed size did not drive an increase in yield as the larger seed size was offset by the lower seed number per plant, to maintain yield levels (Fig. 2). This is expected as similar results were seen in rice using the rice actin promoter and only when *OsBG1* was driven by its own promoter were biomass and yields increased [52]. Other recent attempts to increase yield in wheat by increasing grain size have also been hampered by the negative association between seed size and seed number [53–55]. Even efforts by recurrent selection for higher seed size in wheat breeding programs showed that increases of up to 32 % in seed size were possible but this increase was completely offset by reductions in seed number keeping yields static [56]. However one more recent transgenic approach has been able to overcome this problem by increased expression of an α-expansin from a grain specific promoter [57]. It is interesting that without the increased biomass an increase in overall yield is not seen which may suggest that seed size *per se* is the not the most limiting yield factor and that during flowering the plant can switch between alternative pathways which affect seed number and yields. Further investigation into the spatial and temporal expression patterns of both *TaBG1* and *OsBG1* may help to elucidate how small changes in auxin signaling promote growth and ultimately yield.

As N is a major driver of increased yields and does lead to increased biomass, any increase in yields without an alteration in nitrogen supplied would also increase NUE further driving agronomic improvement [58]. Interestingly no change in the size of the grains were seen in the seeds with increased N treatments. WT size did seem to trend toward larger seeds, but the differences were not significant among the treatments tested and might have been partially offset by the increased seed number with increasing N levels supplied (Fig. 2). To understand the interaction between increase size grain NUE we set out to study the effects that larger grains could have on both the protein content and elemental concentrations of larger grains. It was found that under moderate N levels increasing grain size significantly decreased the protein content of the grain. This was the only level of N for which a difference could be seen and may suggest that increased N is again more important in helping to drive biomass gains leading to increased yields and more specific N treatments are necessary to understand the overall effects of protein content. This could also lead to lower NUE as yields are not increased and overall N in the grain is lower. This did not unfortunately lead to a higher NER which would sacrifice N in the grain to increase yields however this was not seen in any of the overexpression lines.

The increased seed size also led to significantly lower Zn levels in the grain but not other micronutrients such as Fe or Mn. It has been previously shown that altered auxin levels can lead to lower Zn levels [59, 60]. One hypothesis is that this is due to the lower ratio of aleurone ells to endosperm cells resulting in a different in micronutrient density [36]. To a large extent similar patterns were identified here as P, Zn and K were lower in most of the over expression lines. This is most likely explained by the distribution in the wheat grain as Zn and P are in the aleurone layer but elements such as K are stored in the embryo. Other elements more widely distributed like S and Mg showed no clear trends suggesting that lower tissue types can explain the variation in most elements except Fe [61].

## Conclusions

We have identified the orthologues of BG1 genes in wheat and demonstrated that alteration in expression of one of these genes can lead to significantly larger grains. However constitutive expression of *TaBG1* alone will not increase overall yield as the plant compensates for the larger seed size by producing fewer grains. Other tradeoffs from this increased seed size are the change in the nutritional profile of the seeds, with lowered Zn and P levels but not Fe, S or Mg and potentially lower protein content under moderate application of N. Manipulation of *TaBG1* alone increased seed size significantly. However, this increase in seed size is not sufficient to increase yields in wheat but could be a target for further breeding efforts in combination with other genes for increased biomass. Further work to evaluate whether the expression of *TaBG1* or *OsBG1* expressed from their native promoters may compensate for the reduced grain number to increase yield in wheat should also be considered.

## Supplementary Information

**Additional file 1.**

**Additional file 2.**

## Data Availability

Data and seed are available upon request from the corresponding author. Seed materials will be transferred under MTA.

## Abbreviations

BG1: Big Grain 1
N: Nitrogen
Zn: Zinc
P: Phosphate
Fe: Iron
S: Sulphur
K: Potassium
Mn: Manganese
WT: Wild Type
NUE: Nitrogen Use Efficiency
NER: Nitrogen Efficiency Ratio
Ca: Calcium
Mg: Magnessium
B: Boron.
TGW: Thousand Grain Weight
cDNA: complementary DNA
