# Supplementary material for: Ectopic expression of TaBG1 increases seed size and alters nutritional characteristics of the grain in wheat but does not lead to increased yields
Source: BMC Plant Biol. 2021 Nov 10;21:524. doi: 10.1186/s12870-021-03294-x (PMC8579524; doi:10.1186/s12870-021-03294-x)
Supplement: Supplementary file 2 — Additional file 2. [file 12870_2021_3294_MOESM2_ESM.docx]

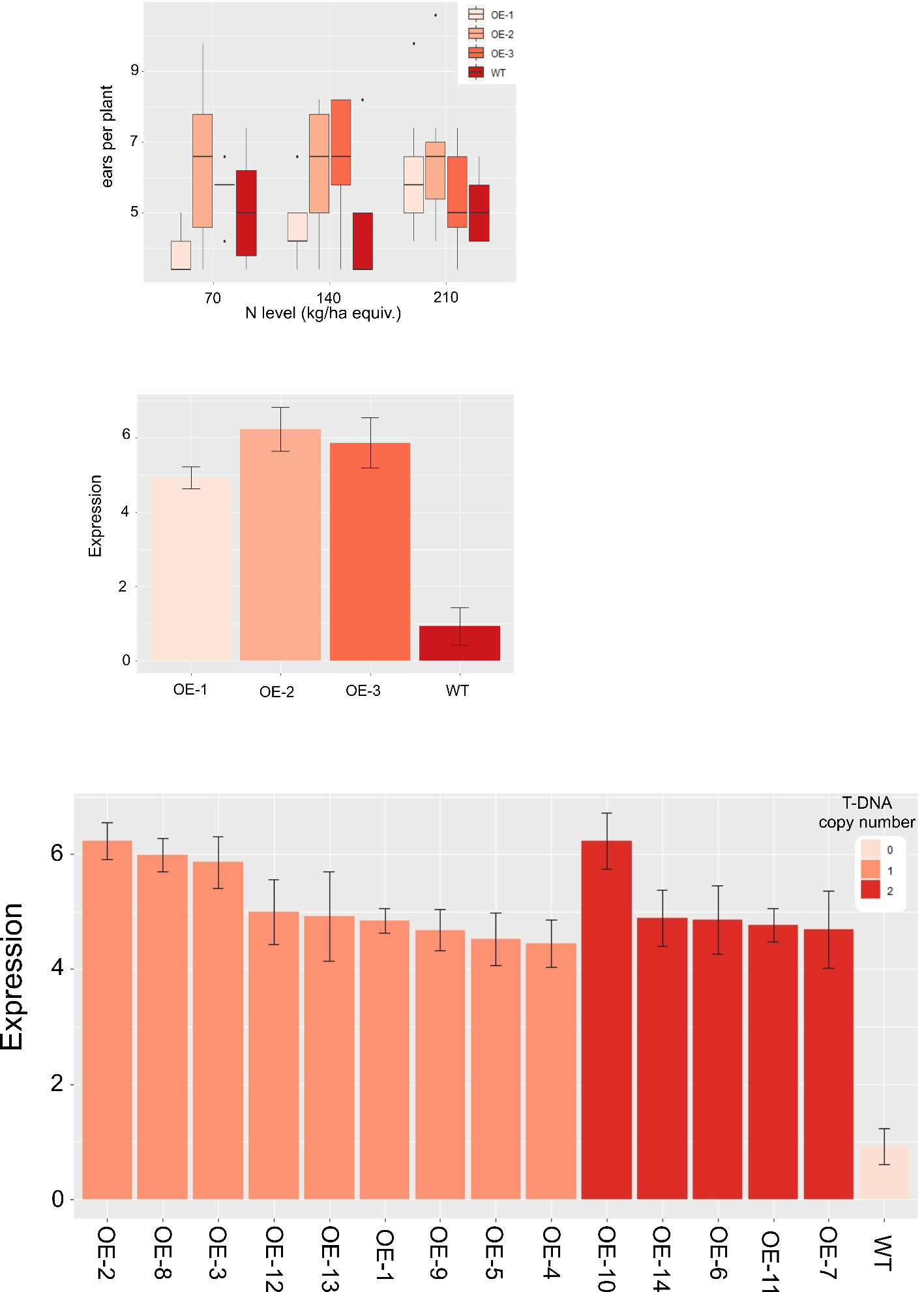


Suppl. Figure 1: Expression of *TaBG1* in shoots of T_0_ plants overexpressing *TaBG1-A* in wheat containing one or two T-DNA insertions, as measured by *nptII* copy number. Expression values shown are relative to the expression of *TaUbi.*


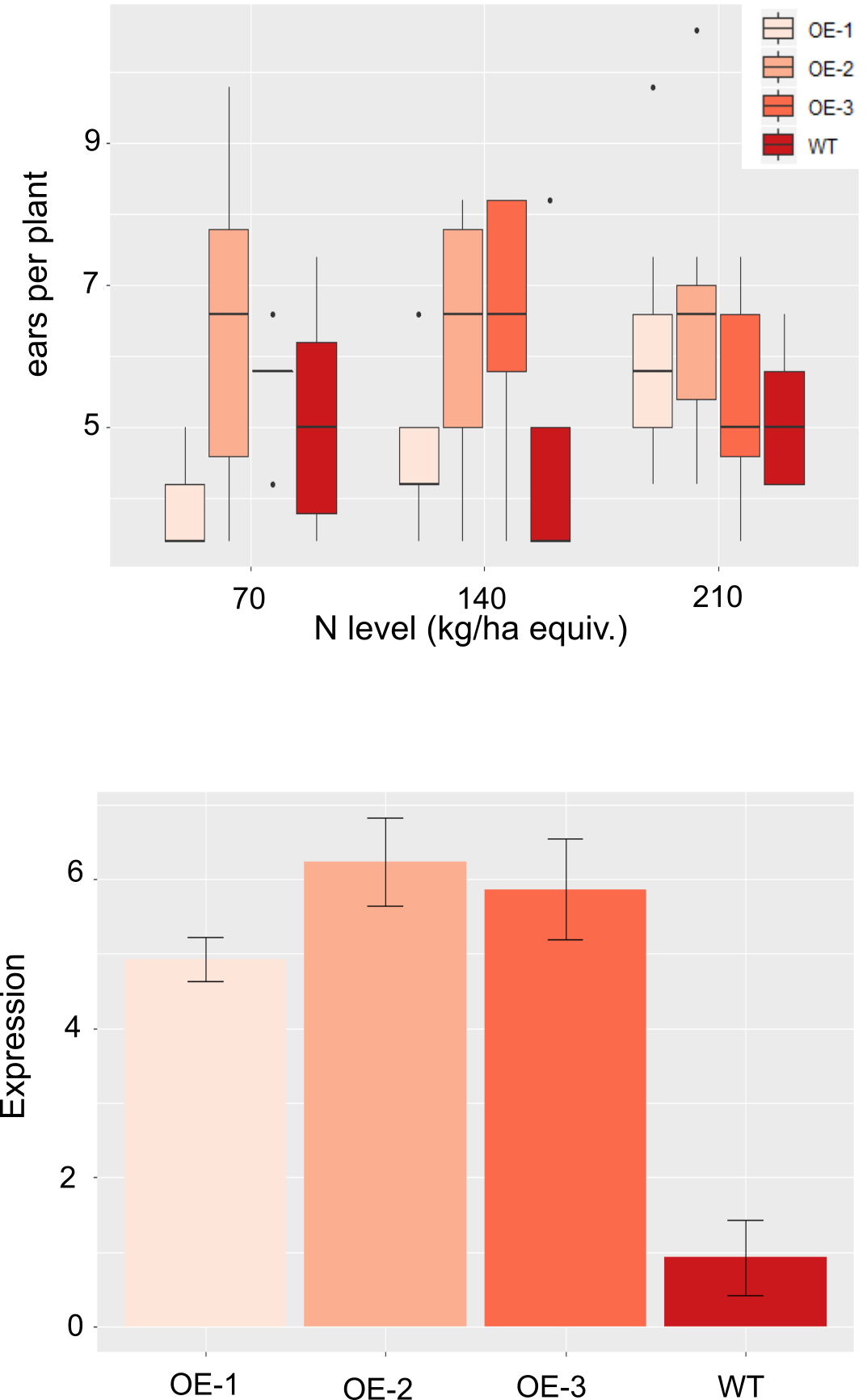


Suppl Figure 2: Number of tillers from plants overexpressing of TaBG1-A. Shown are the measurements from twenty-one plants under three levels of nitrogen, low (70), sufficient (140), and high (210) levels of N. No significant difference to WT were observed.


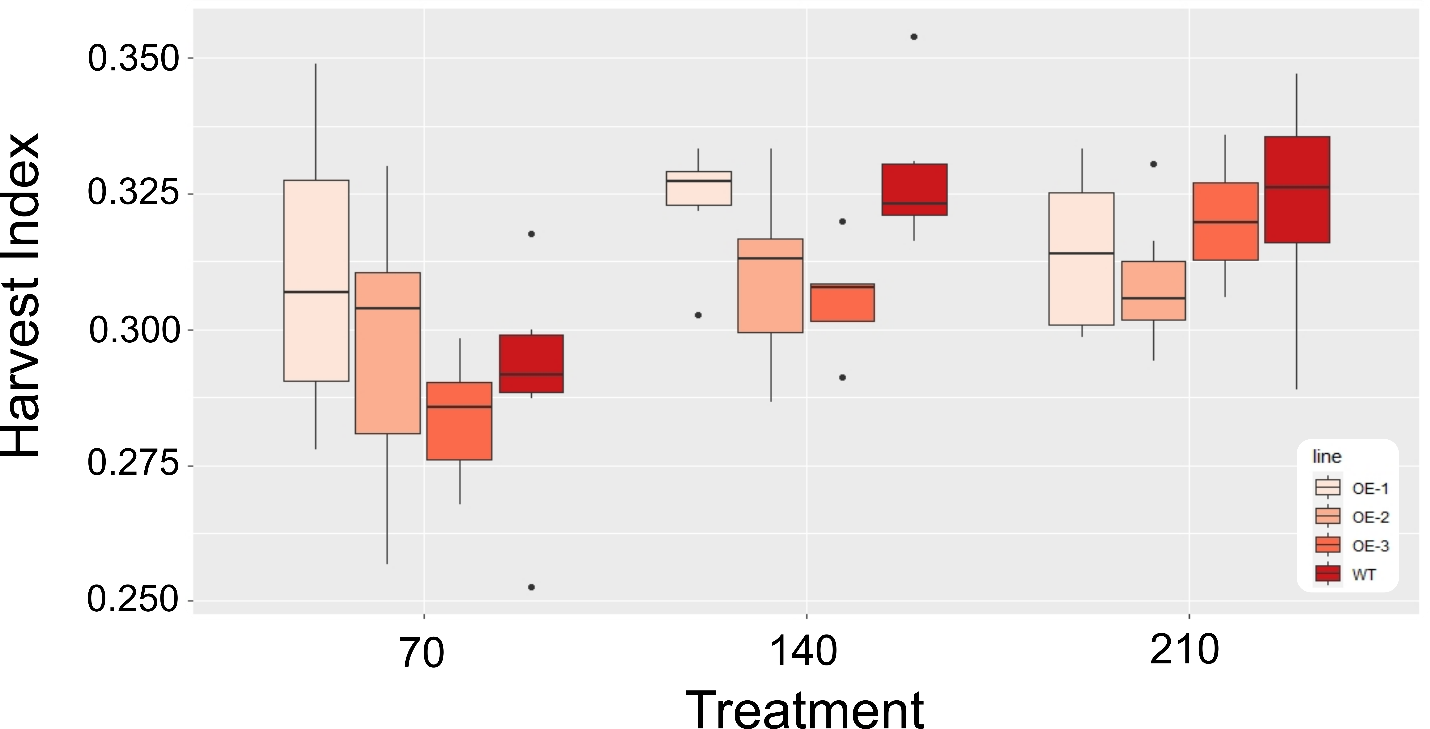


Suppl Figure 3: Harvest index of three transgenic lines relative to a null segregant (WT) grown under three different N levels.


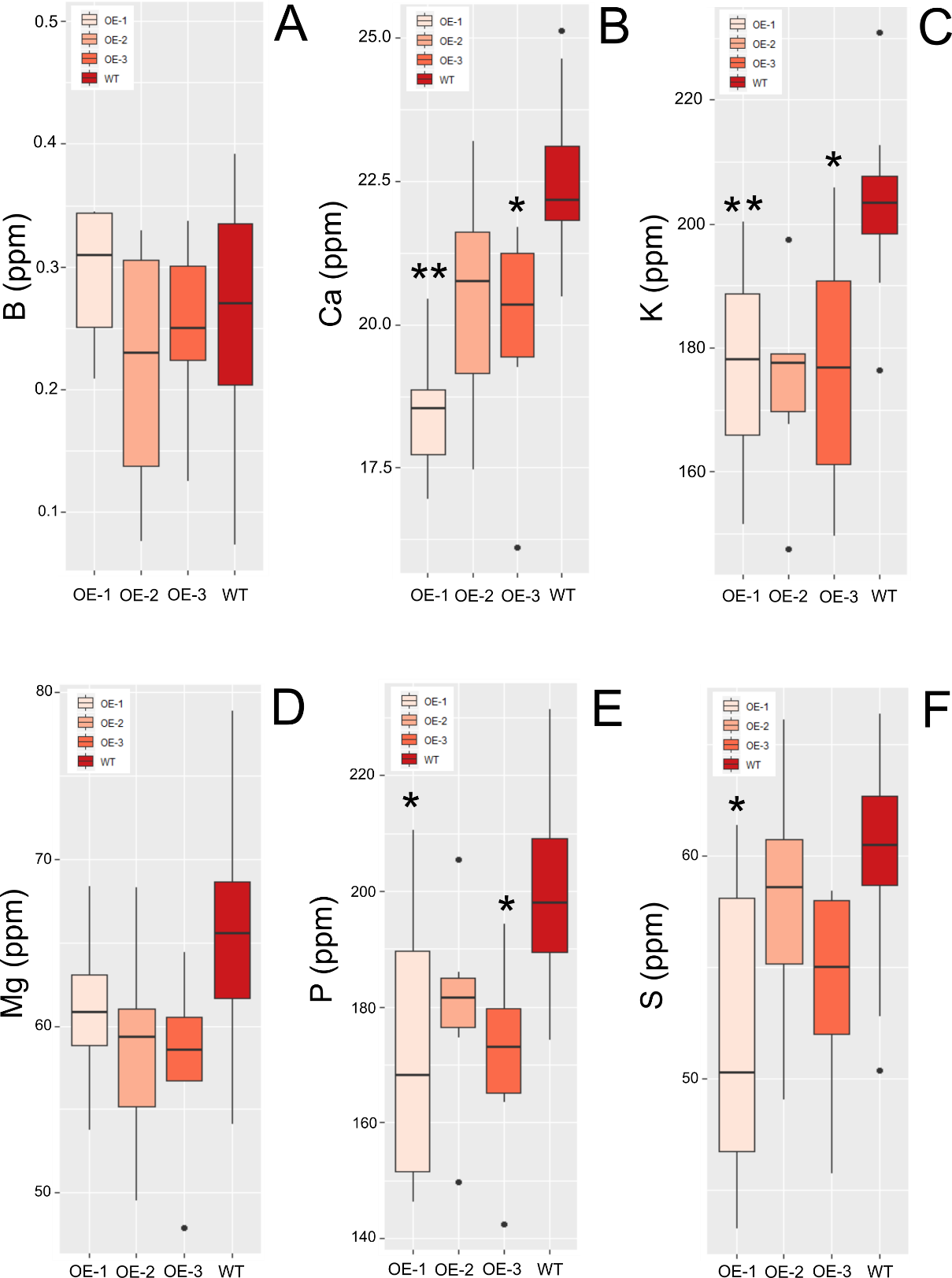


Suppl. Figure 4: Elemental content of TaBG1-A overexpression lines. A) B, B) Ca, C) K, D) Mg, E) P, F) S levels in grains of TaBG1-A overexpression lines relative to WT Fielder. * indicates a significant difference relative to WT p val < 0.05, **p val < 0.01.


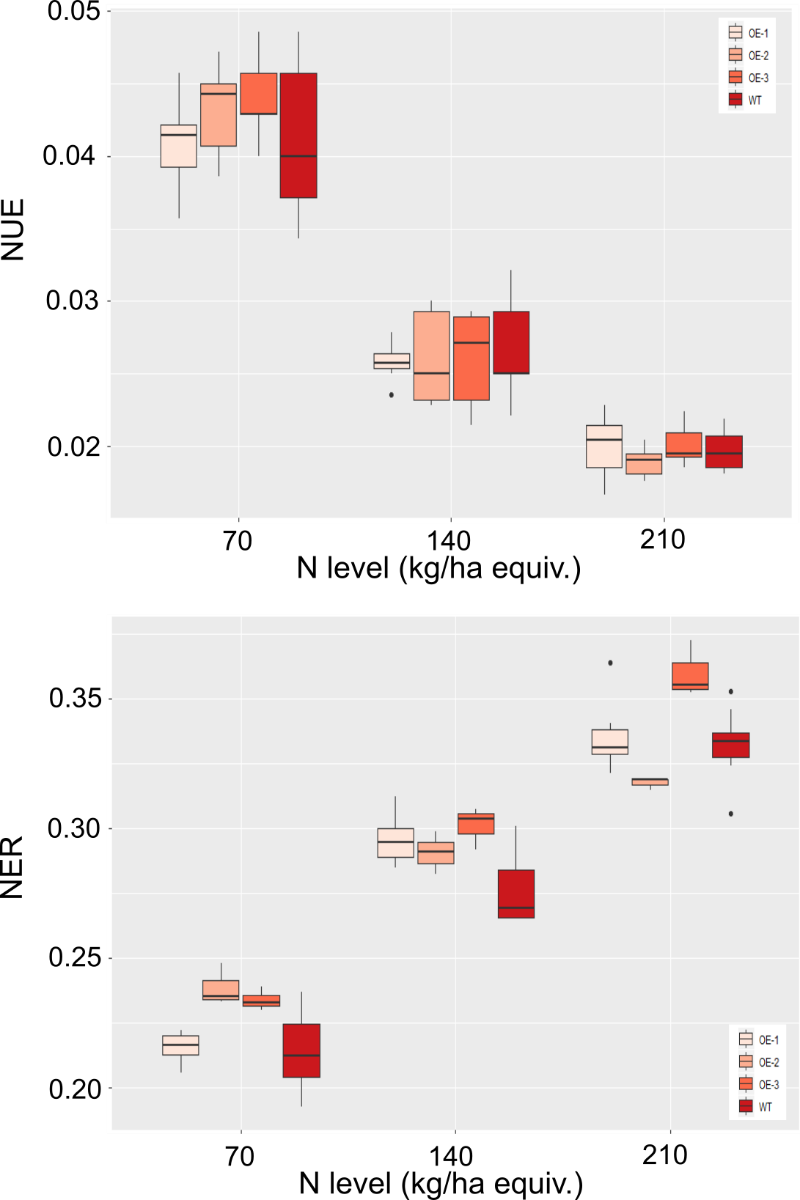


Suppl. Figure 5: Nitrogen Use Efficiency and Nitrogen Efficiency Ratio of TaBG1-A overexpression lines grown under three different N levels. No significant difference to WT was observed.
